# Prevalence of psychiatric disorders for Indigenous Australians: a population-based birth cohort study

**DOI:** 10.1017/S204579602100010X

**Published:** 2021-03-10

**Authors:** James M. Ogilvie, Stacy Tzoumakis, Troy Allard, Carleen Thompson, Steve Kisely, Anna Stewart

**Affiliations:** 1 Griffith Criminology Institute, Griffith University, Mount Gravatt, QLD 4122, Australia; 2 School of Criminology and Criminal Justice, Griffith University, Mount Gravatt, QLD 4122, Australia; 3 School of Medicine, The University of Queensland, Woolloongabba, Australia; 4 Departments of Psychiatry, Community Health and Epidemiology, Dalhousie University, Halifax, NS B3H 4R2, Canada

**Keywords:** Indigenous people, inequalities, mental health, record linkage, substance use disorders

## Abstract

**Aims:**

Limited information exists about the prevalence of psychiatric illness for Indigenous Australians. This study examines the prevalence of diagnosed psychiatric disorders in Indigenous Australians and compares this to non-Indigenous Australians. The aims were to: (1) determine prevalence rates for psychiatric diagnoses for Indigenous Australians admitted to hospital; and (2) examine whether the profile of psychiatric diagnoses for Indigenous Australians was different compared with non-Indigenous Australians.

**Methods:**

A birth cohort design was adopted, with the population consisting of 45 141 individuals born in the Australian State of Queensland in 1990 (6.3% Indigenous). Linked administrative data from Queensland Health hospital admissions were used to identify psychiatric diagnoses from age 4/5 to 23/24 years. Crude lifetime prevalence rates of psychiatric diagnoses for Indigenous and non-Indigenous individuals were derived from the hospital admissions data. The cumulative incidence of psychiatric diagnoses was modelled separately for Indigenous and non-Indigenous individuals. Logistic regression was used to model differences between Indigenous and non-Indigenous psychiatric presentations while controlling for sociodemographic characteristics.

**Results:**

There were 2783 (6.2%) individuals in the cohort with a diagnosed psychiatric disorder from a hospital admission. The prevalence of any psychiatric diagnosis at age 23/24 years was 17.2% (491) for Indigenous Australians compared with 5.4% (2292) for non-Indigenous Australians. Indigenous individuals were diagnosed earlier, with overrepresentation in psychiatric illness becoming more pronounced with age. Indigenous individuals were overrepresented in almost all categories of psychiatric disorder and this was most pronounced for substance use disorders (SUDs) (12.2 *v.* 2.6% of Indigenous and non-Indigenous individuals, respectively). Differences between Indigenous and non-Indigenous Australians in the likelihood of psychiatric disorders were not statistically significant after controlling for sociodemographic characteristics, except for SUDs.

**Conclusions:**

There is significant inequality in psychiatric morbidity between Indigenous and non-Indigenous Australians across most forms of psychiatric illness that is evident from an early age and becomes more pronounced with age. SUDs are particularly prevalent, highlighting the importance of appropriate interventions to prevent and address these problems. Inequalities in mental health may be driven by socioeconomic disadvantage experienced by Indigenous individuals.

Australian Aboriginal and Torres Strait Islander Peoples have experienced significant and compounding disadvantage since British colonisation.^1^ Indigenous Australians are estimated to represent 3.3% of the Australian population (Australian Bureau of Statistics, 2019) and experience disproportionate levels of poor social and health outcomes when compared with their non-Indigenous counterparts (Vos *et al*., 2009; Australian Institute of Health and Welfare [AIHW], 2015). Specifically, Indigenous Australians experience elevated rates of mental health service usage (AIHW, 2020*b*), psychological distress (Jorm *et al*., 2012; AIHW, 2015), alcohol and/or substance use (O'Leary *et al*., 2013) and hospitalisation rates for self-harm and suicide (Dickson *et al*., 2019). Despite well documented poor mental health outcomes for Indigenous Australians, there are no reliable baseline prevalence rates of specific psychiatric disorder diagnoses for this population. The aim of this study is to establish baseline prevalence rates of diagnosed psychiatric disorders from hospital admissions for Indigenous Australians in a birth cohort of individuals born in the state of Queensland in 1990.

A narrative systematic review examining prevalence rates of psychiatric disorders for Indigenous Australians (*N* = 17) found significant methodological variation across studies, resulting in wide discrepancies in prevalence rates (Black *et al*., 2015). Elevated rates (up to ≈50% in some instances) of psychiatric disorder in Indigenous individuals were present for psychotic disorders, major depression, post-traumatic stress disorder, anxiety and alcohol or substance dependence (Black *et al*., 2015). Importantly, prison and detention settings were most commonly used to derive samples (*n* = 8), with only three community-based samples, including one population-based design of new/expectant mothers (i.e. O'Leary *et al*., 2013). Since incarcerated samples have significantly elevated rates of psychiatric illness compared with the general community (Fazel and Seewald, 2012), derived estimates of the prevalence of psychiatric disorders in Indigenous Australians may be inflated. Studies that utilised community samples were either unrepresentative or methodologically weaker. Furthermore, no study compared rates with non-Indigenous groups, limiting conclusions about relative prevalence rates for Indigenous and non-Indigenous individuals. This information is essential for policymakers when identifying specific areas of mental health needs for Indigenous groups and appropriately distributing resources.

Recent studies have examined the prevalence of psychiatric disorders using samples more representative of the wider Indigenous Australian community (Nasir *et al*., 2018; Gynther *et al*., 2019). Nasir *et al*. (2018) reported higher 12-month (2.4 times) and lifetime (1.3 times) rates for psychiatric disorder among Indigenous Australians convenience sampled from Aboriginal medical services compared with the general Australian population, with comorbid psychiatric disorders three to four times higher. Rates of common psychiatric disorder for Indigenous individuals in remote areas were half that of individuals living in urban or regional locations (Nasir *et al*., 2018). However, this contrasts with the findings of Gynther *et al*. (2019) relating to treated psychotic disorders, where high prevalence rates were evident in remote North Queensland Indigenous communities. These conflicting results suggest that prevalence rates of psychiatric illness may vary across diagnosis and specific communities/locales. Indeed, Indigenous Australians are culturally, geographically and sociologically diverse, highlighting the importance of examining the impact of residential location on psychiatric disorder prevalence rates in Indigenous populations.

There are limited representative population-based studies about the extent of diagnosed psychiatric illness for Indigenous Australians (e.g. O'Leary *et al*., 2013, Lima *et al*., 2019). Lima *et al*. (2019) examined the prevalence of mental health-related contacts for mothers of Indigenous children born between 1990 and 2013 in Western Australia. In the cohort, 34% of Indigenous children were born to a mother who experienced a mental health contact either 5 years before birth or 1 year after birth. Mental health contacts were most prevalent for substance-related disorders, mood disorders, anxiety and broader mental health-related concerns. Living in more disadvantaged areas was associated with a higher risk of being born to a mother with a mental health contact. However, these findings relate to service contacts and not actual diagnoses, highlighting the need for population-based studies examining rates of diagnosed psychiatric illnesses.

In summary, accurate and generalisable estimates on rates of psychiatric disorders in Indigenous Australians are not available (Black *et al*., 2015, 2017) and this is consistent with the state of international research for other Indigenous groups (Nelson and Wilson, 2017). Research on psychiatric morbidity among Indigenous Australians is primarily based on specialised at-risk samples (i.e. prisoners), limiting generalisability to the broader Indigenous population. There are also disparate findings on the sociodemographic profiles of Indigenous individuals more likely to experience psychiatric illness (e.g. rural/remote residence), highlighting the importance of population-based studies to establish prevalence rates for different groups of individuals. The current study aims to estimate baseline prevalence rates of diagnosed psychiatric disorders from hospital admissions for Indigenous Australians using a population-based birth cohort of individuals born in Queensland in 1990 and followed up to age 23/24 years. Furthermore, potential psychiatric and sociodemographic profile differences between Indigenous and non-Indigenous individuals that receive psychiatric diagnoses will be explored.

## Method

### Data sources and study design

A population-based birth cohort design was used to examine the prevalence of diagnosed psychiatric disorders among Indigenous and non-Indigenous Australians. Data were derived from the Queensland Cross-sector Research Collaboration (QCRC) repository (see Stewart *et al*., 2015), which contains all individuals born in the state of Queensland in 1990 who have experienced contact with the following Queensland Government administrative systems and data custodians: Queensland Registry of Births, Deaths and Marriages (RBDM); Queensland Department of Child Safety, Youth and Women (child protection); Queensland Department of Youth Justice; Queensland Department of Justice and Attorney General (adult courts and corrections) and Queensland Health. Data across these systems were linked by the Queensland Government Statistician's Office (QGSO), except for Queensland Health datasets that were de-identified and linked within the agency. Probabilistic data linkage methods were used to link individuals across datasets, with identifying information removed before being released to researchers. Data are stored in the Social Analytics Lab, which is a secure purpose-built facility for storing sensitive data (Stewart *et al*., 2015).

The 1990 Queensland birth cohort comprises 45 223 individuals, including 2852 (6.3%) Indigenous individuals. Queensland is the third most populous state in Australia, comprising about 20% of the Australian population with 5.1 million residents (ABS, 2020). At the 2016 census, Indigenous individuals represented 4.0% of the total Queensland population (ABS, 2017). Queensland is ethnically and geographically diverse, covering an area of over 1.8 million km^2^ (ABS, 2017). Approximately half of the population resides in the state capital, the remainder is relatively decentralised throughout the state. In comparison, 62.1% of Indigenous Queenslanders live outside of the state capital, predominately in regional centres.

Based on Queensland RBDM data, there were 266 deaths recorded for the cohort (representing 0.6%), with 24.1% of these deaths occurring between birth and 3-years-old. Indigenous Australians were significantly overrepresented in cohort deaths (*χ*^2^(1, *N* =  45 223) = 7.36, *p* < 0.01, *ϕ*_c_ = 0.01). Cases with deaths before 8-years-old were excluded (*n* = 80), given that the youngest age for first diagnosis in the cohort was 8 years. A further two cases were excluded due to missing age at death, resulting in a final cohort of 45 141 individuals for analysis.

Ethical approval was obtained from the Griffith University Human Research Ethics Committee (HREC 2010/479), and approval was granted by relevant government data custodians. The Strengthening the Reporting of Observational Studies in Epidemiology guidelines were adopted for this study (von Elm *et al*., 2008).

### Measures of psychiatric diagnoses

The Queensland Hospital Admitted Patient Data Collection (QHAPDC) data held by the QCRC was used to measure psychiatric diagnoses for the cohort. Queensland Health linked, de-identified and then appended QGSO's unique person identifier to this dataset before being provided directly to Griffith University. This dataset includes information about hospital admissions in Queensland that directly or indirectly relate to psychiatric diagnoses. All diagnoses are classified according to the International Statistical Classification of Diseases and Related Health Problems – tenth edition Australian modification (ICD-10AM; World Health Organization, 2004) diagnostic system. QHAPDC data are available from July 1995 until extraction on 30/06/2014, with data therefore available from age 4/5 to 23/24 years for the QCRC cohort.

Individuals were classified as having a mental illness if they had ever experienced a hospital admission and received a psychiatric diagnosis (ICD-10 *Mental and behavioural disorders* [codes F00 to F99], as well as suicidal ideation [code R45.8] and self-harm [codes X60 through X84]) as either a primary or additional diagnosis. All diagnoses across all admission episodes were included for individuals up to the date of extraction. Therefore, the data represent lifetime prevalence of diagnosed mental illness from hospital admissions up to age 23/24 years. Psychiatric diagnoses were classified into six broad categories, with each broad category further subdivided into more detailed subcategories (see online Supplementary Table S1 for detailed subdivisions and corresponding ICD-10 codes):

1. Severe mental disorders
2. Common mental disorders
3. Personality disorders
4. Substance use disorders (SUDs) (excludes substance-induced psychoses, which are included under severe mental disorders)
5. Other adult onset disorders
6. Other childhood onset disorders

Diagnostic categories and subdivisions contained mutually exclusive sets of ICD-10 codes and were coded as binary indicators (present/not present) for each individual. Individuals could appear in more than one diagnostic category/subdivision if they received diagnoses across different psychiatric categories/subdivisions.

### Covariates

Eight presentation-related and sociodemographic factors recorded in the QHAPDC dataset were considered as covariates in analyses comparing psychiatric diagnostic profiles of Indigenous and non-Indigenous individuals:

1. Sex (male/female).
2. Age in years at first hospital admission involving a psychiatric diagnosis.
3. Number of hospital admissions involving a psychiatric diagnosis.
4. Length of stay in days at first hospital admission involving a psychiatric diagnosis.
5. Remoteness of usual place of residence at first admission: coded according to the Australian Standard Geographical Classification – Remoteness Area classification (ABS, 2018); with three classes for univariate analyses (major cities/inner regional, outer regional and remote/very remote) and two classes for multivariate analyses (remote/very remote *v.* all others).
6. Index of relative socio-economic advantage and disadvantage for usual place of residence at first admission, derived from the Socio-Economic Indexes for Areas (ABS, 2011). Lower scores indicate greater disadvantage and lack of advantage, while higher scores indicate a relative lack of disadvantage and greater advantage.
7. Dual diagnosis, defined as the presence of a SUD diagnosis with another psychiatric diagnosis (present/not present).
8. Comorbidity, defined as the presence of at least two psychiatric diagnoses from different subcategories excluding SUD diagnoses (present/not present).

### Statistical analyses

Analyses were conducted using R version 3.6 (R Core Team, 2019) in four stages. First, the prevalence (to age 23/24 years) and cumulative incidence (from four/five to 23/24 years) of psychiatric disorder diagnosis for the cohort were stratified by Indigenous status. Prevalence was defined as the proportion of the cohort with any psychiatric disorder diagnosis from a hospital admission by age 23/24 years. Cumulative incidence was defined as the cumulative proportion of the cohort receiving their first psychiatric diagnosis by age 23/24. Cumulative incidence of first diagnosis by age was modelled using the survival package for R (Therneau, 2015). For deceased cases, age at death was taken as the point of censoring for the cumulative incidence analysis.

Second, crude prevalence rates by diagnostic divisions and subdivisions are presented, along with bivariate *χ*^2^ tests to explore differences across Indigenous status. Third, differences in psychiatric presentation and sociodemographic covariates for Indigenous and non-Indigenous individuals are examined using a series of *χ*^2^ and *t*-tests. Fourth, logistic regression was performed to examine differences in the psychiatric diagnosis and sociodemographic profiles of Indigenous and non-Indigenous individuals. Indigenous status was the outcome variable, with the eight covariates and six broad categories of psychiatric disorder entered as predictors. Some individuals were missing data on the covariates of length of stay at first admission (*n* = 27), the index of relative socio-economic advantage and disadvantage (*n* = 32) and remoteness of residence (*n* = 32). Analyses involving these covariates were conducted excluding these cases.

## Results

Of the 45 141 individuals in the cohort, 6.5% (*n* = 2937) had experienced at least one hospitalisation involving a psychiatric diagnosis by age 23/24 (Table 1).

**Table 1.** Crude lifetime prevalence of hospital admissions with psychiatric diagnoses by Indigenous status up to age 23/24 years (*N* =  45 141)

	Indigenous [*n* (%)]	Non-Indigenous [*n* (%)]	Total	*χ*^2^a^^	*ϕ*_c_
Hospital admission with a psychiatric diagnosis	523 (18.3%)	2414 (5.7%)	2937 (6.5%)	699.03***	0.12
Cohort	2851 (6.3%)	42 290 (93.7%)	45 141		

a Pearson's *χ*^2^ test with Yates correction, df = 1; *ϕ*_c_ = Cramer's *V* effect size for *χ*^2^ test.

*p < 0.05, **p < 0.01, ***p < 0.001.

There were statistically significant differences in hospital-derived psychiatric diagnosis prevalence rates based on Indigenous status (Table 1). Indigenous individuals compared with non-Indigenous individuals had higher rates of hospitalisations involving any psychiatric diagnosis (18.3 *v.* 5.7%; *χ*^2^(1, *N* =  45 141) = 699.03, *p* < 0.001, *ϕ*_c_ = .12). Indigenous individuals receiving a psychiatric diagnosis were more likely than non-Indigenous individuals to be admitted to hospital through emergency departments (see online Supplementary Table S2 for details on hospital admission source).

**Fig. 1.**
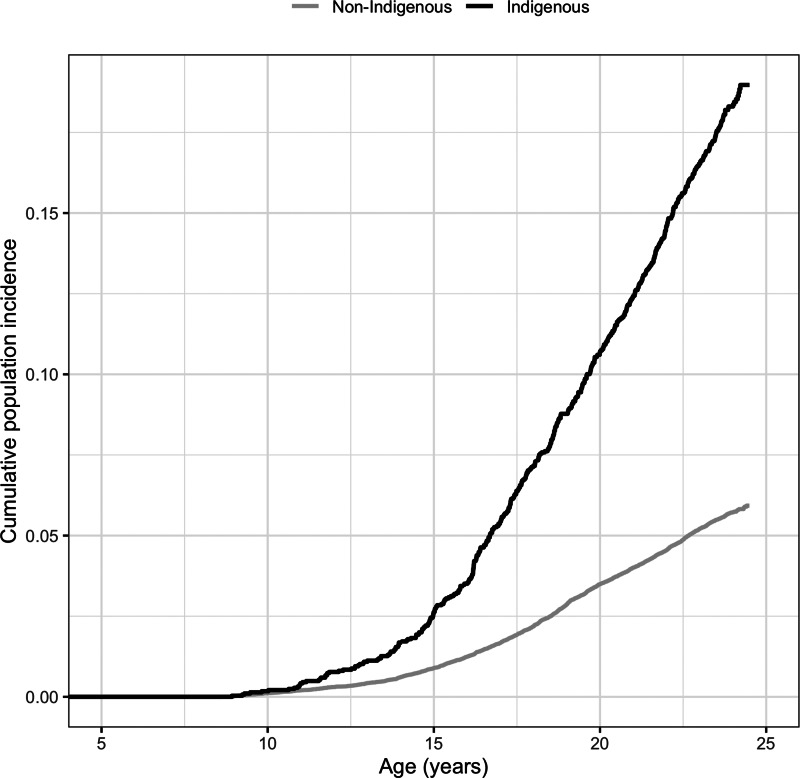
Cumulative incidence of any psychiatric diagnosis from a hospital admission up to age 23/24 years by Indigenous status.

The cumulative incidence by age for individuals receiving their first psychiatric diagnosis is illustrated in Fig. 1. There was a statistically significant difference in cumulative incidence rates from age 4/5 to 23/24 years between Indigenous and non-Indigenous individuals, confirmed by a significant Gray's (1988) test (*χ*^2^(1, 45 141) = 644.30, *p* < 0.001). For Indigenous individuals, the incidence of psychiatric diagnoses occurred at an earlier age and at a faster rate over the age period when compared with non-Indigenous individuals.

Cohort prevalence rates were examined by broad and subdivisional categories of psychiatric diagnosis and separated by Indigenous status (Table 2). At the broad classification level, Indigenous individuals were significantly overrepresented in all categories of psychiatric disorder, although the effect sizes were small in most cases. Indigenous overrepresentation was most pronounced for SUDs, where 12.2% of Indigenous individuals had received an SUD diagnosis by age 23/24, compared with 2.6% of non-Indigenous individuals. This was mainly driven by high rates of diagnosed alcohol use disorders among Indigenous individuals (9.4%) compared with non-Indigenous individuals (2.0%).

**Table 2.** Crude population lifetime prevalence (to age 23/24) of psychiatric diagnoses from hospital admissions by Indigenous status (*N* = 45 141)

Psychiatric diagnosis	Indigenous [*n* (%)]^a^	Non-Indigenous [*n* (%)]^a^	Total	*χ*^2^b^^	*ϕ*_c_
Any disorder	523 (18.3%)	2414 (5.7%)	2937 (6.5%)	699.03***	0.12
Severe	78 (2.7%)	440 (1.0%)	518 (1.1%)	66.20***	0.04
Schizophrenia, schizoaffective and other psychotic disorders	51 (1.8%)	146 (0.3%)	197 (0.4%)	124.80***	0.05
Severe or psychotic affective disorders	28 (1.0%)	295 (0.7%)	323 (0.7%)	2.66	0.01
Psychotic disorders related to substance use	24 (0.8%)	83 (0.2%)	107 (0.2%)	44.38***	0.03
Common	161 (5.6%)	931 (2.2%)	1092 (2.4%)	132.88***	0.05
Depressive and other mood disorders	74 (2.6%)	479 (1.1%)	553 (1.2%)	46.04***	0.03
Phobic anxiety disorders	12 (0.4%)	134 (0.3%)	146 (0.3%)	0.60	>0.01
Reaction to severe stress	71 (2.5%)	292 (0.7%)	363 (0.8%)	106.23***	0.05
Adjustment disorders	48 (1.7%)	283 (0.7%)	331 (0.7%)	36.38***	0.03
Other anxiety disorders	62 (2.2%)	411 (1.0%)	473 (1.0%)	36.12***	0.03
Personality	38 (1.3%)	214 (0.5%)	252 (0.6%)	31.42***	0.03
Cluster A	<5	^c^	10 (0.0%)	–	
Cluster B	31 (1.1%)	161 (0.4%)	192 (0.4%)	29.84***	0.03
Cluster C	<5	^c^	15 (0.0%)	–	
Other personality	11 (0.4%)	63 (0.1%)	74 (0.2%)	–	
Substance use	348 (12.2%)	1116 (2.6%)	1464 (3.2%)	776.05***	0.13
Alcohol	267 (9.4%)	846 (2.0%)	1113 (2.5%)	599.35***	0.12
Other substance	156 (5.5%)	463 (1.1%)	619 (1.4%)	375.11***	0.09
Other adult onset	189 (6.6%)	864 (2.0%)	1053 (2.3%)	244.58***	0.07
Organic disorders	10 (0.4%)	48 (0.1%)	58 (0.1%)	–	
Eating disorders	<5	^c^	85 (0.2%)	–	
Self-harm	170 (6.0%)	748 (1.8%)	918 (2.0%)	233.72***	0.07
Other Adult onset disorders	14 (0.5%)	44 (0.1%)	58 (0.1%)	–	
Other child onset	55 (1.9%)	334 (0.8%)	389 (0.9%)	39.26***	0.03
Mental retardation	24 (0.8%)	91 (0.2%)	115 (0.3%)	38.84***	0.03
Disorders of psychological development	15 (0.5%)	153 (0.4%)	168 (0.4%)	1.53	0.01
Childhood behavioural	23 (0.8%)	130 (0.3%)	153 (0.3%)	18.27***	0.02
Other childhood onset disorders	13 (0.5%)	69 (0.2%)	82 (0.2%)	11.07***	0.02

a Cells with less than five individuals are presented as <5 to preserve the anonymity of the individuals.

b Pearson's *χ*^2^ test with Yates correction, df = 1; ‘–’ denotes *χ*^2^ unable to be estimated due to low cell numbers; *ϕ*_c_ = Cramer's *V* effect size for *χ*^2^ test.

c Complimentary cell suppression to prevent cell count calculation from marginal totals.

*p < 0.05, **p < 0.01, ***p < 0.001.

Indigenous individuals were also overrepresented in almost all subdivisions of psychiatric disorders such as schizophrenia and other psychotic disorders (1.8 and 0.3% for Indigenous and non-Indigenous respectively), self-harm (6.0 *v.* 2.0%); and reaction to severe stress disorders (2.5 *v.* 0.5%), which includes posttraumatic stress disorder.

There were statistically significant differences in the sociodemographic characteristics of individuals with a psychiatric diagnosis when stratified by Indigenous status (Table 3). When compared with non-Indigenous individuals, Indigenous individuals with a psychiatric diagnosis were more likely to reside in locations characterised by higher levels of disadvantage and in outer regional and remote/very remote locations; and be diagnosed with a dual disorder. In contrast, when compared with Indigenous individuals, non-Indigenous individuals with a psychiatric diagnosis were more likely to reside in major cities or inner regional locations and more likely to have comorbid psychiatric diagnoses.

**Table 3.** Sociodemographic and psychiatric presentation characteristics of cohort individuals with a psychiatric diagnosis by Indigenous status (*N* = 2937)

Characteristic	Indigenous	Non-Indigenous	Total	Group difference (*t*/*χ*^2^)^a^	Effect size (*ϕ*_c_ /*d*)
Sex				2.68	0.03
Female	230 (44.0%)	1160 (48.1%)	1390 (57.6%)		
Male	293 (56.0%)	1255 (52.0%)	1548 (64.1%)		
Age at first hospitalisation [mean (s.d.)]	18.38 (3.40)	18.20 (3.50)	18.23 (3.48)	1.11	0.05
Number of admissions [mean (s.d.)]	2.72 (6.96)	2.75 (8.00)	2.75 (7.83)	−0.09	−0.00
Length of stay first admission [mean (s.d.)]^b^	3.83 (8.61)	4.63 (9.19)	4.49 (9.09)	−1.82	−0.09
Index of relative disadvantage for residence [mean (s.d.)]^c^	3.67 (2.32)	5.30 (2.58)	5.01 (2.61)	−13.19***	−0.64
Remoteness of residence [*n* (%)]^d^					
Major cities/inner regional	255 (48.8%)	1946 (80.6%)	2201 (74.9%)	235.48***	0.28
Outer regional	155 (29.6%)	385 (15.9%)	540 (18.4%)	53.44***	0.14
Remote/very remote	106 (20.3%)	58 (2.4%)	164 (5.6%)	258.02***	0.30
Dual diagnosis [*n* (%)]	163 (31.2%)	502 (20.8%)	665 (22.6%)	25.86***	0.09
Comorbid diagnoses [*n* (%)]	152 (29.1%)	917 (38.0%)	1069 (36.4%)	14.36***	0.07

a Indigenous status differences examined using Pearson's *χ*^2^ test (df = 1) with Yates correction for categorical variables; and independent samples *t-*test (df *=* 2936) for continuous variables; *ϕ*_c_ = Cramer's *V* effect size for *χ*^2^ test; *d* = Cohen's *d* effect size for *t*-test.

b Excluding *n* = 27 cases with unknown length of stay.

c Excluding *n* = 32 cases with unknown index values.

d Excluding *n* = 32 cases with unknown residence.

*p < 0.05, **p < 0.01, ***p < 0.001.

The logistic regression model was significant (*χ*^2^ = (14, *N* = 2905) = 376.95, *p* < 0.001) indicating that psychiatric and sociodemographic profiles differentiated Indigenous from non-Indigenous individuals with a psychiatric diagnosis (see online Supplementary Table S3 for correlations of variables in the model). The model explained between 14% (McFadden pseudo-*R*^2^) and 20% (Cragg–Uhler pseudo-*R*^2^) of the variance and correctly classified 83.5% of cases. Results for individual factors (Table 4) highlighted two significant sociodemographic variables; specifically, Indigenous individuals with a psychiatric diagnosis were more likely to reside in remote locations and locations with higher levels of disadvantage compared with non-Indigenous individuals. When considering the psychiatric and sociodemographic factors altogether, it was evident that there were no significant differences between Indigenous and non-Indigenous individuals in the prevalence rates of all psychiatric diagnoses except for SUDs. Individuals with a SUD diagnosis were two times more likely to be Indigenous than non-Indigenous.

**Table 4.** Logistic regression results for sociodemographic and psychiatric profile differences between Indigenous and non-Indigenous individuals with a psychiatric diagnosis (*N* = 2905^a^)

				95% CI for OR		
Variable	*B*	s.e.	OR	Lower	Upper	Wald *χ*^2^
Sex	0.03	0.11	1.03	0.83	1.27	0.06
Age at first hospitalisation	0.01	0.02	1.01	0.98	1.05	0.65
Number of admissions	0.01	0.01	1.01	0.99	1.02	0.62
Length of stay first admission	0.01	0.01	1.00	0.99	1.01	0.03
Index of relative disadvantage for residence	−0.22	0.02	0.80	0.76	0.84	100.30***
Remoteness of residence	1.20	0.11	3.31	2.67	4.10	118.26***
Dual diagnosis	0.25	0.19	1.28	0.88	1.87	1.70
Comorbid diagnoses	−0.34	0.18	0.71	0.50	1.02	3.58
Severe disorder	−0.15	0.18	0.86	0.60	1.24	0.65
Common disorder	−0.06	0.16	0.94	0.69	1.27	0.17
Personality disorder	0.12	0.22	1.12	0.72	1.74	0.27
SUD	0.58	0.17	1.79	1.29	2.48	12.27***
Adult onset disorder	0.28	0.15	1.32	0.98	1.77	3.41
Child onset disorder	0.05	0.20	1.05	0.71	1.55	0.06

*B,* unstandardised coefficient; CI, confidence interval; OR, odds ratio; s.e., standard error of coefficient.

a Logistic regression model estimated from a reduce sample size due to missing data on some covariates.

*p < 0.05, **p < 0.01, ***p < 0.001.

## Discussion

Population-based information on the prevalence of psychiatric disorders for Indigenous people is vital for strengthening mental health policies and to better understand the mental health needs of Indigenous Peoples internationally. This study compared prevalence rates of diagnosed psychiatric disorders from hospital admissions for Indigenous and non-Indigenous Australians using administrative health records for a population-based cohort of 45 141 individuals. Four key findings emerged: (1) Indigenous people were overrepresented across most psychiatric disorders, particularly SUDs; (2) Indigenous overrepresentation in psychiatric diagnosis begins in childhood and becomes more pronounced in adulthood; (3) there are important sociodemographic differences between Indigenous and non-Indigenous individuals who receive psychiatric diagnoses from hospital admissions and (4) the increased likelihood of Indigenous individuals being diagnosed across all categories of psychiatric disorder except for SUDs disappeared after accounting for sociodemographic and other psychiatric-related variables. Overall, a very clear picture of Indigenous overrepresentation in psychiatric morbidity emerged from the data. By age 23/24 years, Indigenous Australians were diagnosed with psychiatric disorders at a rate over three times that of non-Indigenous Australians (172.16 *v.* 54.12 per 1000). The high rate of psychiatric disorders among Indigenous Australians is consistent with survey evidence, as well as available statistics demonstrating overrepresentation in most areas of the public health system (Cunningham and Paradies, 2012; Jorm *et al*., 2012; AIHW, 2015, 2018, 2020*b*).

The elevated prevalence of SUDs among Indigenous Australians in our study is consistent with previous research (Teesson *et al*., 2000; AIHW, 2006; Wilkes *et al*., 2014), as is the elevated rate of dual diagnosis (i.e. SUDs combined with other psychiatric disorders; Young *et al*., 2018). Over one-quarter of Indigenous individuals with a psychiatric diagnosis presented with a dual diagnosis. The relationship between substance use and mental illness is understood to be bidirectional, where substance use may elevate the risk of and/or increase the severity of psychiatric illnesses, while psychiatric illness may also increase the likelihood of problematic substance use as a dysfunctional coping mechanism (Siegfried, 1998). Furthermore, the co-occurrence of psychiatric and SUDs is strongly linked to poor patient outcomes, reflecting the extent of difficulties faced by this group (Dixon *et al*., 2016). International evidence also highlights that SUDs are strongly associated with the co-occurrence of other and often multiple chronic health conditions (e.g. diabetes, chronic kidney disease and ischaemic heart disease), mental health conditions (Siegfried, 1998) and a heightened risk of hospitalisation (Wu *et al*., 2018; Young *et al*., 2018). From a public health perspective, the identification of an SUD during hospitalisation may represent an opportunity to intervene to address co-occurring physical and mental health difficulties for Indigenous Australians. It is possible that chronic health conditions experienced by Indigenous Australians bring them into increased contact with the hospital system, where psychiatric diagnoses are made.

Our findings confirm previous research that mental health and substance use problems emerge at an early age for Indigenous individuals (AIHW, 2011, 2015, 2020*a*). It was evident that Indigenous overrepresentation started at an earlier age and accelerated at a greater rate up to age 23/24 years when compared with non-Indigenous individuals. This resulted in more pronounced disparities as the cohort aged, which underscores the importance of early intervention among vulnerable individuals in Indigenous communities. The early onset of psychiatric disorder diagnosis for Indigenous Australians likely reflects the extreme levels of multiple and accumulated disadvantage faced by Indigenous Australians from an early age that is particularly harmful to psychological health (Twizeyemariya *et al*., 2017) and potentially contributes to the emergence or exacerbation of other social and health problems. For example, substance use is a key risk factor for involvement in criminal offending (Morgan *et al*., 2013), which may contribute to the overrepresentation of Indigenous youth in the criminal justice system (White, 2014).

Despite the obvious need for interventions to address substance use problems among Indigenous youth, there is a limited evidence-base about what works in reducing substance use and addressing the negative effects of substance use problems (including other mental health needs) for this population (Zubrick *et al*., 2010; Geia *et al*., 2018). Although it is important to deliver interventions that are culturally responsive and appropriate, it is also important not to lose sight of addressing the underlying factors driving problematic substance use for Indigenous individuals (e.g. intergenerational trauma, social inequality and socioeconomic disadvantage). Without an effective understanding of the factors associated with substance use problems among Indigenous Australians, policies and interventions to address these problems are likely to be simplistic and ineffective (Wilkes *et al*., 2014). Services and interventions need to be developed and operate in a way that acknowledges the complexity of factors leading to poor mental health for Indigenous Australians.

International evidence highlights the importance of the social environment in shaping health outcomes, with extensive evidence that the most socioeconomically disadvantaged individuals exhibit the worst outcomes across a range of health issues, including mental health (Wilkinson and Marmot, 2003; Marmot, 2005). Indigenous Australians are more likely to experience greater social disadvantage (e.g. poor access to and quality of health care, housing instability, unemployment, low educational attainment and poverty) and adverse life events (e.g. discrimination, child maltreatment, violent victimisation, trauma, grief and loss), which in turn leads to increased levels of psychological distress and the development of psychiatric illness (Zubrick *et al*., 2010). Our findings support this argument that social disadvantage is the driving force behind Indigenous overrepresentation in psychiatric illness, since after adjusting for sociodemographic covariates, differences between Indigenous and non-Indigenous individuals in the likelihood of being diagnosed with the six broad categories of psychiatric disorder disappeared, except for SUDs. From a policy perspective, this highlights the critical importance of social change efforts to address the wider social inequalities experienced by Indigenous Australians and reduce the burden of psychiatric illness.

Indigenous individuals with a psychiatric diagnosis were more likely than non-Indigenous individuals with a psychiatric diagnosis to reside in remote locations. In this study, 20.5% of Indigenous individuals who received a psychiatric diagnosis resided in remote or very remote locations, compared with only 2.5% of non-Indigenous individuals. In contrast, evidence from Western Australia suggested that the prevalence of psychiatric illness and psychological distress was lower for Indigenous individuals residing in remote locations (Zubrick *et al*., 2005; Lima *et al*., 2019). Zubrick *et al*. (2005) proposed that for those living in remote and isolated regions, adherence to Indigenous culture and traditional lifestyles may be protective against developing clinically significant emotional or behavioural difficulties. It is also possible that the impact of remoteness on mental health varies across specific remote locations due to a range of factors (e.g. access to appropriate services and extent of disadvantage). Our findings may reflect unique geographical and cultural issues specific to remote locations in Queensland. Hunter (2007) argued that limited access to services and the presence of entrenched social disadvantage in remote settings are leading issues contributing to the greater burden of mental health problems for some Indigenous populations. In the absence of adequate services to address mental health, episodes of psychiatric illness may be more frequent and/or acute, requiring hospitalisation.

Our findings should be interpreted considering the strengths and limitations of the study. The most notable strength of this study was the use of a population-based birth cohort, increasing the generalisability of prevalence estimates of psychiatric illness. The cohort was large enough to examine specific psychiatric disorders, where previous studies considered broad groupings. Diagnoses were derived from a relatively reliable source (i.e. health practitioners in a hospital setting), increasing the validity of diagnostic estimates. However, the use of hospitalisations as the source of diagnoses is also the most notable limitation. Not all individuals experiencing a psychiatric illness will experience or require hospitalisation, and as a result the data were not able to provide information about individuals experiencing psychiatric illness that is managed in community settings. Therefore, the current prevalence rates should be considered conservative and underestimate the true extent of psychiatric illness. Rates of hospitalisations will differ across disorders, with more severe and/or acute psychiatric illnesses more likely to result in hospitalisation. Indigenous overrepresentation could diminish when taking more common disorders into account. Indigenous individuals may also be reluctant to utilise health services that are not compatible with cultural approaches to health and wellbeing. Additionally, there are potential biases in how Indigenous individuals with psychiatric illnesses utilise are treated within the health system, impacting rates of hospitalisation and diagnosis. For example, the greater prevalence of SUDs for Indigenous individuals may not be a true reflection of the extent of these problems in the population since it is possible that health professionals are more likely to ask about and record substance use as problematic for Indigenous compared with non-Indigenous individuals (O'Leary *et al*., 2013). Finally, the design of the current study did not allow for examination of the temporal order of onset for substance use and mental health problems.

In conclusion, there is clear inequality in psychiatric morbidity between Indigenous and non-Indigenous Australians across most forms of psychiatric illness that is evident from childhood, becomes more pronounced with age and appears to be driven largely by social disadvantage. Indigenous Australians residing in remote locations appeared particularly vulnerable to the emergence of mental health problems, and SUDs were particularly elevated, even when controlling for sociodemographic factors. Therefore, to reduce the gap between Indigenous and non-Indigenous Australians in mental health outcomes, social inequality and disadvantage must continue to be addressed through sustained social change efforts and prioritisation in the long-term political agenda. Moreover, the identified early age of onset for psychiatric diagnosis highlights that mental health services may need to developed and prioritised in childhood and adolescence. Mental health service gaps in remote areas also need to be addressed, with an emphasis on culturally appropriate and strength-based interventions. Finally, integrated substance use and mental health interventions should form a priority in the development of programmes for Indigenous Australians.

## Data Availability

The data for the study are held in Social Analytics Lab (SAL) at Griffith University. Due to privacy, ethical and legal considerations, the QCRC data cannot be shared without direct approval from relevant data custodians and QGSO. Any researcher interested in accessing the data can submit an application to the SAL management committee (socialanalyticslab@griffith.edu.au) with the relevant support and approvals.

## Footnotes

1 In this paper, we respectfully refer to Aboriginal and Torres Strait Islander Peoples as Indigenous Australians or Indigenous people.

## Supplementary material

For supplementary material accompanying this paper visit http://dx.doi.org/10.1017/S204579602100010X. click here to view supplementary material
