# Supplementary material for: Prevalence of psychiatric disorders for Indigenous Australians: a population-based birth cohort study
Source: Epidemiol Psychiatr Sci. 2021 Mar 10;30:e21. doi: 10.1017/S204579602100010X (PMC8061141; doi:10.1017/S204579602100010X)
Supplement: Supplementary file 1 [file S204579602100010Xsup001.docx]

Supplementary Table S1.

Psychiatric disorder diagnostic category classifications by ICD-10-AM codes.

| **Broad Diagnostic Group** | **Detailed Diagnostic Categories** | **ICD-10 Codes** |
| --- | --- | --- |
| **Severe Mental Disorders** | Schizophrenia, schizoaffective and other psychotic disorders | F20, F22, F23, F24, F25, F25.0, F25.1, F25.2, F25.8, F25.9, F28, F29 |
|  | Severe or Psychotic Affective Disorders | F30, F31, F32.2, F32.3 |
|  | Psychotic Disorders related to Substance Use | F10.5, F11.5, F12.5, F13.5, F14.5, F15.5, F15.50, F15.51, F15.59, F15.70, F16.5, F17.5, F18.5, F19.5, F19.7 |
| **Common Mental Disorders** | Depressive and other mood disorders (e.g., recurrent depressive disorder, cyclothymia, dysthymia) | F32.0, F32.1, F32.8, F32.9, F33.0, F33.1, F33.4, F33.8, F33.9, F34, F38, F39 |
|  | Phobic anxiety disorders | F40, F40.1, F40.2, F40.8, F40.9 |
|  | Reaction to severe stress (e.g., acute stress reaction, post-traumatic stress disorder) | F43.0, F43.1, F43.8, F43.9 |
|  | Adjustment disorders | F43.2 |
|  | Other anxiety disorders (e.g., obsessive-compulsive, dissociative and somatoform disorders) | F41, F42, F44, F45, F48 |
| **Personality Disorders** | Cluster A | F21, F60.0, F60.1 |
|  | Cluster B | F60.2, F60.3, F60.30, F60.31, F60.4 |
|  | Cluster C | F60.5, F60.6, F60.7 |
|  | Other Personality Disorders | F60.8, F60.09, F60.9, F61, F62, F62.0, F62.1, F62.8, F62.9, F68.0, F68.1, F68.8, F69 |
| **Substance Use Disorders** | Mental and behavioural disorders due to use of alcohol | F10 |
|  | Mental and behavioural disorders due to use of other substances | F11, F12, F13, F14, F15 F16, F17, F18, F19 |
| **Other Adult Onset Disorders** | Organic disorders (e.g., dementia, disorders due to brain damage or dysfunction, amnesic syndrome) | F00, F01, F02, F03, F04, F05, F06, F07, F09 |
|  | Eating disorders | F50 |
|  | Self-harm and suicidal ideation | R45.8, X60-X84 |
|  | Other Adult onset disorders (e.g. sleep disorders, sexual dysfunction, postnatal and abuse of non-dependence-producing substances, habit and impulse disorders, gender identity disorders, sexual preference disorders; unspecified disorders) | F51, F52, F53, F54, F55, F59, F63, F64, F65, F66, F99 |
| **Other Childhood Onset Disorders** | Mental retardation | F70, F71, F72, F73, F78, F79 |
|  | Disorders of psychological development (e.g., disorders of speech and language, pervasive developmental disorders) | F80, F81, F82, F83, F84, F88, F89 |
|  | Childhood behavioural (e.g., conduct and hyperkinetic disorders, mixed disorders of conduct and emotion) | F90, F91, F92 |
|  | Other childhood onset disorders (e.g., emotional disorders, disorders of social functioning, tic disorders) | F93, F94, F95, F98 |

Supplementary Table S2.

Source of first admission to hospital for individuals receiving a psychiatric diagnosis by Indigenous status and sex (*n* = 2,783).

| Source of admission | Indigenous (n (%)) | Non-Indigenous (n (%)) | Total | χ^2†^ | φ*_c_* |
| --- | --- | --- | --- | --- | --- |
|  |  |  |  |  |  |
| Emergency department | 403  (82.1%) | 1,747  (76.2%) | 2,150  (77.3%) | 7.56** |  |
| Outpatient department | 33  (6.7%) | 214  (9.3%) | 247  (8.9%) | 3.11 |  |
| Other hospital or health facility | 28  (5.7%) | 103  (4.5%) | 131  (4.7%) | 1.06 |  |
| Law enforcement agency or correctional facility | 6  (1.2%) | 16  (0.7%) | 22  (0.8%) | -- |  |
| Private medical practitioner | 9  (1.8%) | 135  (5.9%) | 144  (5.2%) | 12.75*** |  |
| Other | 12  (2.4%) | 77  (3.4%) | 89  (3.2%) | 0.82 |  |

^†^ Pearson’s chi-squared test, *df* = 1; “--” denotes chi-square unable to be estimated due to low cell numbers; φ*_c_* = Cramer’s V effect size for chi-squared test.

** p* <.05, ** *p* <.01, *** *p* <.001

Supplementary Table S3.

Correlations (Pearson’s r) among variables included in the logistic regression.

|  | 1 | 2 | 3 | 4 | 5 | 6 | 7 | 8 | 9 | 10 | 11 | 12 | 13 | 14 |
| --- | --- | --- | --- | --- | --- | --- | --- | --- | --- | --- | --- | --- | --- | --- |
| 1. Indigenous status |  |  |  |  |  |  |  |  |  |  |  |  |  |  |
| 2. Sex | 0.04 |  |  |  |  |  |  |  |  |  |  |  |  |  |
| 3. Age first admission | 0.02 | 0.06* |  |  |  |  |  |  |  |  |  |  |  |  |
| 4. Total psychiatric admissions | 0.00 | -0.09*** | -0.08*** |  |  |  |  |  |  |  |  |  |  |  |
| 5. First length of stay | -0.03* | 0.02 | -0.04 | 0.11*** |  |  |  |  |  |  |  |  |  |  |
| 6. Remote residence | 0.29*** | 0.05* | -0.01 | -0.04* | -0.05*** |  |  |  |  |  |  |  |  |  |
| 7. Disadvantage index | -0.24*** | -0.03 | 0.06** | 0.03 | 0.06*** | -0.25*** |  |  |  |  |  |  |  |  |
| 8. Severe disorders | -0.03 | -0.02 | 0.08*** | 0.26*** | 0.27*** | -0.05** | 0.05* |  |  |  |  |  |  |  |
| 9. Common disorders | -0.06** | -0.13*** | -0.07*** | 0.16*** | 0.08*** | -0.07*** | 0.04* | 0.06* |  |  |  |  |  |  |
| 10. Other adult onset disorders | 0.00 | -0.18*** | -0.07** | 0.15*** | 0.01*** | -0.02 | 0.02 | 0.09*** | 0.30*** |  |  |  |  |  |
| 11. Child onset disorders | -0.04 | 0.07** | -0.39*** | 0.16*** | 0.06** | -0.07*** | -0.04* | 0.02 | -0.01 | -0.05 |  |  |  |  |
| 12. Personality disorder | -0.02 | -0.11*** | 0.00 | 0.33*** | 0.09*** | -0.09*** | 0.06** | 0.28*** | 0.20*** | 0.19*** | 0.07** |  |  |  |
| 13. SUD | 0.16*** | 0.20*** | 0.18*** | 0.02 | -0.11*** | 0.10*** | -0.07*** | -0.02** | -0.24*** | -0.20*** | -0.19*** | 0.05 |  |  |
| 14. Dual diagnosis | 0.09*** | 0.06*** | 0.02 | 0.15*** | 0.07*** | 0.01 | -0.03 | 0.27*** | 0.22*** | 0.25*** | 0.03* | 0.25*** | 0.54*** |  |
| 15. Comorbid diagnoses | -0.07** | -0.13*** | -0.13*** | 0.23*** | 0.13*** | -0.12*** | 0.05** | 0.35*** | 0.55*** | 0.48*** | 0.22*** | 0.36*** | -0.18*** | 0.28*** |

SUD = substance use disorder

** p* <.05, ** *p* <.01, *** *p* <.001
